# Chronic Kidney Disease and Cancer: Inter-Relationships and Mechanisms

**DOI:** 10.3389/fcell.2022.868715

**Published:** 2022-05-18

**Authors:** Mengsi Hu, Qianhui Wang, Bing Liu, Qiqi Ma, Tingwei Zhang, Tongtong Huang, Zhimei Lv, Rong Wang

**Affiliations:** ^1^ Department of Nephrology, Shandong Provincial Hospital Affiliated to Shandong First Medical University, Jinan, China; ^2^ Department of Nephrology, Shandong Provincial Hospital, Cheeloo College of Medicine, Shandong University, Jinan, China

**Keywords:** chronic kidney disease, cancer, tumor, correlation, onco-nephrology, interdiscipline

## Abstract

Chronic kidney disease (CKD) has been recognized as an increasingly serious public health problem globally over the decades. Accumulating evidence has shown that the incidence rate of cancer was relatively higher in CKD patients than that in general population, which, mechanistically, may be related to chronic inflammation, accumulation of carcinogenic compounds, oxidative stress, impairment of DNA repair, excessive parathyroid hormone and changes in intestinal microbiota, etc. And in patients with cancer, regardless of tumor types or anticancer treatment, it has been indicated that the morbidity and incidence rate of concomitant CKD was also increased, suggesting a complex inter-relationship between CKD and cancer and arousing increasing attention from both nephrologists and oncologists. This narrative review focused on the correlation between CKD and cancer, and underlying molecular mechanisms, which might provide an overview of novel interdisciplinary research interests and the potential challenges related to the screening and treatment of CKD and cancer. A better understanding of this field might be of help for both nephrologists and oncologists in the clinical practice.

## Introduction

CKD is defined as abnormalities of kidney structure or function present for >3 months, with implications for health (Kidney Disease: Improving Global Outcomes Glomerular Diseases Work, 2013). Globally, the incidence of chronic kidney disease (CKD) and death due to CKD have increased by 89 and 98%, respectively, from 1990 to 2016, suggesting that CKD has become an increasingly serious public health problem (Xie et al., 2018). In recent years, there has been a remarkable rise in non-cardiovascular mortality in patients with CKD, although in the past, cardiovascular diseases were widely considered as one of the major complications of CKD (de Jager et al., 2014). A large cohort study of CKD from the United Kingdom showed that malignant tumor might be one of the leading causes of non-cardiovascular death in patients with CKD stage G3-G5, accounting for 15% of all deaths in this population (Marks et al., 2013). And thanks to early detection of cancer and novel therapies, the number of cancer survivors has significantly risen, whereas this population might also be suffering a high risk of CKD (Shin et al., 2015). Based on those, onco-nephrology has been put forward, which is a new and fast developing discipline, focusing on the interaction between cancer and kidney disease. In this narrative review, we present the latest findings on the inter-relationship between CKD and cancer and the underlying molecular mechanisms by searching the literature on Pubmed with key words including CKD, cancer and onco-nephrology and article types including reviews, clinical trials, randomised controlled trials and case reports, etc., in order to identify priorities for future work and highlight the urgent need to understand the interaction between CKD and cancer both in basic research and in the clinical setting for nephrologists and oncologists, which might be relevant to improved patient outcome.

## Potential Mechanisms of Increased Cancer Incidence Rate in CKD Patients

Accumulating studies have indicated an increased risk of cancer in CKD patients compared with that in non-CKD patients (Cengiz, 2002; Wong et al., 2009; Premuzic et al., 2019), which might be associated with the decrease in glomerular filtration rate (GFR) or estimated GFR (eGFR) in CKD (Premuzic et al., 2019; Xu et al., 2019). And cancer has been recognized as an independent risk factor for all-cause mortality in advanced CKD (Chinnadurai et al., 2019), the risk of which may vary, instead of being linear with the stage of CKD (Magee, 2014; Park et al., 2019). End-stage kidney disease (ESKD) is the end stage of CKD, with GFR<15 ml/min/1.73 m^2^ (Kidney Disease: Improving Global Outcomes Glomerular Diseases Work, 2013). Previous cohort studies reported that the incidence and prevalence rate of cancer in patients with ESKD showed an upward trend over the long term (Port et al., 1989; Wang et al., 2015). An analysis of data from the United States, Europe, Australia, and New Zealand on renal and urethral cancer in end-stage dialysis patients showed that increased risk of renal parenchymal cancer in ESKD patients was consistent with loss of renal function and its duration, but not with primary kidney disease or dialysis mode, indicating that high stake of cancer in these patients might be associated with renal failure per se, instead of the potential carcinogenic effects of dialysis therapy (Stewart et al., 2003). Notably, it was found the incidence rate of cancer incidence in renal transplant patients with ESKD tripled that of patients before renal transplantation, which might be attributed to immune suppression and oncogenic virus infection (Vajdic et al., 2006; Cheung and Tang, 2019).

### Chronic Inflammation

The inflammatory state of CKD was multifactorial, including increased susceptibility to infection (especially dialysis pathway-related infections), oxidative stress and acidosis, metabolic changes of adipose tissue, and intestinal disorders (Bloembergen and Port, 1996; Dai et al., 2017; Mihai et al., 2018; Ebert et al., 2020). Persistent low-grade inflammation had been identified as a significant pathological feature of CKD (Ebert et al., 2020). On one hand, inflammatory overload of the kidney caused by increased production of pro-inflammatory cytokines including IL-1β, IL-6, and TNF-α might be manifested as impaired renal excretion function, which might prolong plasma half-life of these pro-inflammatory cytokines and be associated with persistent low-grade inflammation in CKD (Gupta et al., 2012). On the other hand, susceptibility to infection might be one of the significant, easily being overlooked, clinical characteristics of the CKD population. Community-population follow-up study showed a crude incidence of infectious events of 23.6 (95% CI, 22.8-24.6) per 1,000 person-years in CKD patients, with an increased risk of hospitalization for infection in both those on dialysis therapy and those with less severe renal insufficiency who did not require dialysis (Ishigami et al., 2017). Another report indicated that the incidence of common infectious complications in CKD patients who had not yet started dialysis was about 3 times that of the general population (Naqvi and Collins, 2006). And the admission rate due to this infection event in CKD population was 4 times higher than that in non-CKD population, and in dialysis patients it become 10 times higher than non-CKD population (Naqvi and Collins, 2006).

It has been recognized that chronic inflammation was one of the most common features of infection (Kovalchuk et al., 2014). Similarly, inflammation has long been considered as one of the risk factors and one of the important participants in the process of cancer formation, as inflammatory environment might enable tumor cells to escape host immune surveillance, leading to subsequent angiogenesis, tumor growth, invasion, and metastasis (Coussens and Werb, 2002). Also, chronic inflammation was likely to lead to DNA mutation and deregulation and release of carcinogenic cytokines, a key step implicated during the development of cancer (Wong et al., 2012). Chronic inflammation due to infection could directly induce DNA damage through production of nitric oxide derivative nitrogen dioxide by phagocytes, and indirectly by mutagen peroxynitrite generated by the reaction of nitric oxide with superoxide anion (Kovalchuk et al., 2014). The DNA-damaged cells were likely to cause mutations in oncogenes or tumor suppressor genes, resulting in a clonal population of cells with a proliferative advantage (Basu, 2018).

Interestingly, inflammation and oxidative stress might interact with each other and play pivotal roles in CKD. Chen et al. detected significant upregulation of inflammatory factors including cyclooxygenase-2 (COX-2), inducible nitric oxide synthase (INOS) and monocyte chemotactic protein-1 (MCP-1), downregulation of pro-oxidant genes p47phox, and downregulation of antioxidant systems [nuclear factor erythroid related factor 2 (Nrf2), catalase, heme oxygenase 1 (HO-1), glutathione peroxidase (GPx), glutamate transporter protein-1 (GSH-1)] in blood and urine specimens from 180 CKD patients (Chen et al., 2017). And this inflammatory/oxidative process was accompanied by activation of Wnt/β-catenin signaling pathway (Chen et al., 2017). Multiple evidence has indicated that Wnt/β-catenin signaling pathway was involved in the development and progression of solid tumors and hematologic malignancies (Zhang and Wang, 2020). In mice, it was reported that *β*-catenin activated by Wnt pathway synergized with other oncogenic pathways or chemicals to induce liver cancer (He and Tang, 2020). Wnt/β-catenin signaling pathway has also been indicated to regulate differentiation, proliferation, apoptosis and migration of multiple myeloma cells (Hu and Hu, 2018).

Besides, in renal tubular epithelial cells, aldosterone could induce trans-activation of epidermal growth factor receptor (EGFR) via ADAM17, followed by the release of transforming growth factor-α, which regulated the expression of pro-inflammatory factors such as chemokine ligand 2 (CCL-2) and chemokine ligand 5 (CCL-5) (Morgado-Pascual et al., 2015). The pro-inflammatory factors CCL-2 and CCL-5 were reportedly associated with tumor growth, angiogenesis, and invasion of breast cancer, while CCL-2 could indirectly stimulate tumorigenic activity of normal breast cells (Yu et al., 2018; Yamaguchi et al., 2021). CCL-5 secreted by tumor-associated macrophages (TAMs) might promote the progression of prostate, gastric and colorectal cancers. However, the carcinogenic effects of both in CKD need to be further explored in the future (Yamaguchi et al., 2021).

Interestingly, it was found that angiotensin II (Ang-II) might stimulate superoxide production through NAD(P)H oxidase, while angiotensin-converting enzyme inhibitors (ACEI)/angiotensin receptor blockers (ARB) might reduce superoxide production by blocking Ang-II production or by preventing AngII from binding to the receptor (de Cavanagh et al., 2004); and the authors suggested that these drugs might hold an antioxidant effect and might improve the oxidative stress state of diseases (de Cavanagh et al., 2004). However, it seemed to be contradictory to the clinical data that the incidence rate of cancer in CKD patients was relatively higher than non-CKD patients, although ARB/ACEI have been frequently used in CKD patients. This inconsistency might be due to other potential risk factors or molecular mechanisms in CKD patients, which we would discuss later in this review, and might be resulted from the potential effects or impacts of common application of other drugs in these CKD patients such as erythropoietin (EPO) for the treatment of anemia in CRF patients, which has been implicated to be related to promotion of tumor growth with undetermined mechanisms (González Vitores et al., 1999; Ribatti, 2010). These factors might outweigh the potential antioxidant effect of ACI/ARB and further explorations are in need in the complex setting of CKD.

### Accumulation of Carcinogenic Compounds

In ESKD patients, due to impaired renal function, nitrogen-containing substances and carcinogenic compounds accumulated in the blood, which put the body in a uremic environment (Masereeuw et al., 2014). It was found that levels of carcinogenic compounds 2-amino-6-methyldipyrido [1,2-a:3′,2′-d]imidazole (Glu-P-1) and 2-aminodipyrido [1,2-a:3′,2′-d]imidazole (Glu-P-2) were relatively higher in plasma of uremic patients on dialysis than in normal population and levels of both compounds remained high after 1 month of dialysis treatment, indicating that uremic patients might be persistently exposed to high levels of carcinogenic compounds (Manabe et al., 1987). Animal studies had shown that several oncogenic heterocyclic amines, including Glu-P-1, 2-amino-3,8-dimethylimidazo [4,5f]quinoxaline (MeIQx), and 2-amino-3-methylimidazo [4,5-f]quinoline (IQ), might be moderately carcinogenic to the liver, breast and intestine, but limited to dietary acquisition and exposure at certain doses and times (Dooley et al., 1992). Mechanistically, this might be related to activation of these oncogenic chemicals as electrophilic species during metabolism through binding to DNA or causing DNA damage (Basu, 2018). These studies suggested that reduced excretion of partial renal excretion of carcinogenic compounds in renal failure led to accumulation of such substances in plasma, and it was plausible to infer that a certain dose and duration of exposure to carcinogenic compounds in uremic environment might be associated with a high incidence of malignancy in patients with chronic renal failure.

Aromatic hydrocarbon receptor (AHR) was a ligand-activated transcription factor, which was well known for regulating the toxicity of carcinogen “dioxin” and has been implicated in tumorigenesis (Opitz et al., 2011). There were studies showing levels of serum aryl hydrocarbon receptor-activating potential (AHR-AP) were elevated before dialysis but decreased after dialysis in CKD patients (Dou et al., 2018). The authors indicated that in these patients high levels of AHR-AP reflected the accumulation of uremic toxins, the latter of which were AHR agonists, and that AHR might be activated under such conditions (Dou et al., 2018). Animal studies showed 5/6 nephrectomy approach induced an approximately 3-fold increase in serum urea concentration and in AHR-AP in CKD mice compared to the sham-operated group (Dou et al., 2018). AHR could also mediate transcriptional upregulation of cytochrome P450 Phase 1 hydrocylases CYP1A1, CYP1A2 and CYP1B1, the latter of which has been implicated to be a risk factor for the carcinogensis of certain cancers (Lao et al., 2014; Wang Z. et al., 2020). These studies suggested that uremic toxins may be involved in tumorigenesis in CKD through its interaction with activation of this AHR signaling.

### Oxidative Stress

Oxidative stress is a state of imbalance between oxidation and antioxidant action in the body, which has been implicated in CKD, manifested as increased oxidative activity and decreased antioxidant system (Morena et al., 2002; Putri and Thaha, 2014). In early stages of CKD, continuous low and chronic inflammation in CKD patients might trigger the generation of NADPH oxidase and MPO by polymorphonuclear neutrophils and monocyte-macrophages, which promoted formation of reactive oxygen species (ROS) and synergistically participated in the oxidative stress process (Locatelli et al., 2003; Putri and Thaha, 2014). A study of 87 patients with CKD whose plasma levels of the oxidative stress indicator 8-epiPGF2a were significantly increased with the progression of CKD staging showed that oxidative stress levels were increased in late renal insufficiency stage of CKD (Dounousi et al., 2006). At the same time, excess ROS reduced clearance of pro-oxidant substances and decreased antioxidant function presented in patients with CKD would combine to increase oxidative stress levels, and thereby create a pro-oxidant environment (Locatelli et al., 2003). Under physiological conditions, transcription factor Nrf2 played a significant role in antioxidant responses through nuclear factor erythroid 2-related factor 2/antioxidant response element (Nrf2/ARE) signaling pathway (Shaw and Chattopadhyay, 2020), whereas in uremic and hemodialysis patients, the antioxidant effect was reduced (Stockler-Pinto et al., 2018). Upon stimulation by oxidative stress, dissociation of covalently bound nuclear factor erythroid 2-related factor 2-Kelch-like ECH-associated protein1 (Nrf2-keap1) in the cytoplasm allowed free translocation of Nrf2 to the nucleus, where it heterodimerized with Maf (musculoaponeurotic fibrosarcoma oncogene homolog) proteins to form the Nrf2/Maf complex, which subsequently initiated ARE-dependent gene expression of antioxidant and cytoprotective proteins (Tu et al., 2019). In peripheral blood mononuclear cells of uremic and hemodialysis patients, Nrf2-associated antioxidant genes, such as heme oxygenase-1(HO-1), glutamate-cysteine ligase modifier subunit (GCLM) and catalase, were downregulated, whereas NF-κB activation showed opposite effects, leading to upregulated expression of oxidant genes and proteins and increased oxidative stress levels (Pedruzzi et al., 2015; Feng et al., 2019). Activation of NF-κB might be associated with certain T cell lymphoma (Giri and Aggarwal, 1998).

During oxidative stress, ROS was constantly generated by aerobic metabolism in mitochondria, which may contribute to serious damage to cell structure and function and induce somatic mutation and tumor transformation (Loft and Poulsen, 1996; Reuter et al., 2010), including increasing DNA mutations or inducing DNA damage, genomic instability, and malignant cell proliferation (Reuter et al., 2010). In addition, ROS itself was involved in cancer migration as an important component of certain signaling pathways. *In vitro* experiments showed that bladder cancer cell line TSGH-8301 was more dispersed and separated from each other after stimulation with uremic toxin p-cresyl (P-CS), and indicated that ROS was a key signal for cell migration induced by P-CS which further induced bladder cancer cell migration and epithelial mesenchymal transition (EMT) through reactive oxygen species/Src/focal adhesion kinase (ROS/Src/FAK) signaling pathway (Peng et al., 2020). It was indicated that activation of Src could contribute to the growth, survival, migration, and metastasis of malignant tumors (breast, prostate, and lung cancers), as well as FAK, which could also regulate migration and invasion of a variety of cancer cells, under the regulation of ROS signals (Lim et al., 2012).

### Impairment of DNA Repair

Low DNA damage repair capacity was associated with the risk of cancer development and has long been considered a cause of malignancy (Sevilya et al., 2014). Experiments on repair capacity of lymphocytes from normal people or patients with chronic renal failure (CRF) treated with or without dialysis showed that DNA repair capacity of lymphocytes in CRF patients on dialysis was similar to that of normal control population, which, in contrast, was significantly reduced after UV or gamma irradiation in the latter population, suggesting that patients with CRF might have a reduced capacity to repair DNA damage and might regain this capacity through dialysis (Malachi et al., 1993). Unrepaired or improperly repaired DNA damage would lead to mutable genes and aberrations in the chromosome, which might be responsible for cancer susceptibility, that was, the malignant and pathological transformation of cells (Schupp et al., 2010; Helena et al., 2018). Irreparable genomic damage, such as sister chromatid exchanges and chromosome breaks, and abnormal distribution of entire chromosomes, has been found in lymphocytes of dialysis patients, correlated to an increased incidence of cancer in these patients, particularly in kidney, prostate, liver, and uterine cancers and lymphoma (Stopper et al., 1999; Buemi et al., 2006). Polymorphism studies of DNA repair enzyme genes Xeroderma pigmentosum complementation group D (XPD) and X-ray cross-complementing group 1 (XRCC1) in dialysis patients and healthy controls indicated these DNA damage repair variants were significantly associated with susceptibility to the development of ESKD (Trabulus et al., 2012).

### Excessive Parathyroid Hormone

With the gradual decline of renal function, urinary phosphorus excretion was reduced and increased blood phosphorus was increased. Blood phosphorus and calcium combine to form calcium phosphate, which lowered blood calcium. Low blood calcium, high blood phosphorus, and lack of active vitamin D were all recognized as the main causes of secondary hyperparathyroidism (SHPT) (Lau et al., 2018). It had been reported that parathyroid hormone (PTH) and parathyroid hormone receptors stimulated the proliferation of some tumor cells, such as osteoblasts, human renal carcinoma cell and breast cancer cells, indicating excessive parathyroid hormone might be pro-carcinogenic to some extent (Birch et al., 1995). In an experimental study in uremic rats, rats in the sham-surgery group with preserved parathyroid glands showed high PTH and high FGF-23 during renal failure induced by feeding the adenine high-phosphorus diet, compared to control rats with destroyed parathyroid glands, implicating that hyperparathyroidism secondary to CKD could induce increased serum levels of FGF-23 (Lavi-Moshayoff et al., 2010). High expression of FGF-23 in CKD patients might be associated with regulation of FGF-23 production by bone remodeling through release of low molecular weight FGFs (Silver and Naveh-Many, 2013). Although there was a lack of research on tumor-promoting effects of high expression of FGF-23, the important role of FGF/FGFR signaling in prostate cancer and paraneoplastic diseases mediated by FGF23 overexpression, such as hypophosphatemia, also suggested a link between FGF-23 and tumors (Lee et al., 2014).

Deficiency of vitamin D (VD) was often observed in CKD patients (Diniz et al., 2012; Feldman et al., 2014). VD is the precursor to the potent steroid hormone calcitriol (also known as 1,25-dihydroxy- vitamin D3 (1,25(OH)2D3)), widely involved in a series of physiological processes in the body and multiple cellular signaling pathways in relation to cancer risk and prognosis (Jones, 2010). *In vitro* studies suggested some potential anti-tumor mechanisms of calcitriol, one of the active metabolites of VD, including stagnation of tumor cell cycle, promotion of tumor cell apoptosis, inhibition of tumor angiogenesis and invasion, etc (Trump and Aragon-Ching, 2018). Several controlled experimental studies have suggested that DV deficiency (VDD) might be associated with the development of several malignancies (breast, colorectal and bladder, etc) (Palmieri et al., 2006; Jacobs et al., 2013; Chandler et al., 2015). Others demonstrated that VDD might be associated with an increased risk of esophageal squamous cell cancer, oral cancer, and throat cancer, and the expression of VD receptor was increased in precancerous lesions and oral cancers (Botelho et al., 2020). From this point of view, it might be reasonable to expect a potential anti-cancer role of supplementation of VD. And there were some studies implicated that supplementation of VD might be correlated with a reduced risk of cancer (Feldman et al., 2014; Theodoratou et al., 2014). However, a meta analysis concluded that there was still a lack of sufficiently convincing evidence for a significant association between VD and cancer (Theodoratou et al., 2014). And randomized control trials in humans did not yet exist to supportively conclude a beneficial role for VD supplementation in the clinical setting (Feldman et al., 2014). It would be of importance to conduct further experiments and trials to identify the role of VDD and VD supplementation in cancer development and treatment in CKD patients.

In the treatment of secondary hyperparathyroidism, some first-line drug treatments included vitamin D preparations, calcimimetics, etc. For refractory secondary hyperparathyroidism, parathyroidectomy (PTx) might be considered an effective treatment. Observational studies have shown a 34% reduction in all-cause mortality in patients with SHPT treated with PTx compared to patients with SHPT not treated with PTx, suggesting that parathyroidectomy might be beneficial to the survival of CKD patients with SHPT (Komaba et al., 2015). Nevertheless, large observational studies of tumor morbidity and mortality in CKD patients with combined SHPT after surgical treatment are still lacking.

### Changes in Intestinal Microbiota

Uremia might directly or indirectly influence the composition of intestinal microbiota and intestinal barrier (Syed-Ahmed and Narayanan, 2019). Some researchers used antibiotics to eradicate the facultative anaerobic microbiota in the intestinal tract of CKD mice, which effectively prevented bacterial translocation, significantly reduced the levels of serum endotoxin, and completely reversed all markers of systemic inflammation to the level of non-CKD control mice (Andersen et al., 2017). A large number of studies reported that lactobacilli decreased in the intestinal microbiota of CKD animals and patients, while Enterobacteriaceae with a gene encoding tryptophan tyrosine phenol lyase increased, demonstrating they might come into play in production of uremic toxins (Kikuchi et al., 2017).

As mentioned earlier, AHR was a ligand activated transcription factor known for its tumor-promoting effects, and uremic toxins derived from intestinal microbiota have been recognized as effective endogenous ligands for AHR activation (Murray et al., 2014). Other mechanisms have been proposed that the imbalance of intestinal flora might aggravate the development of intestinal tumors and inhibit antitumor immunity, and might be related to the followings: 1) destruction of DNA: This might arise by the injury induced by intestinal flora producing specific toxins like Colibactin; 2) activation of carcinogenic signal pathways, including PI3K/Akt, Wnt and NF-κB signaling pathways, which were activated by the toxins expressed by *Helicobacter pylori*; and 3) production of tumor-promoting metabolites such as secondary bile acids, secreted or produced by coupled binding of bile acids by intestinal microbiota (Mima et al., 2017). Intestinal microbiota also communicated with the brain by regulating levels of some metabolites including tryptophan, 5-hydroxytryptamine and short-chain fatty acids, changed the central higher-order behavior, and indirectly participated in the regulation of body emotion and cognition (Jenkins et al., 2016; Mohajeri et al., 2018). Emotional and cognitive regulation disorders caused by changes in intestinal microbiota might also play a role in mental diseases such as anxiety and depression, which were closely related to cancer (Spiegel and Giese-Davis, 2003; Skonieczna-Zydecka et al., 2018; Wang Y.-H. et al., 2020). Very recently, novel examination method has been developed, such as the addition of fecal F nucleatum quantitation to fecal immunochemical test for screening characteristics of the intestinal microbiome, which greatly improved the sensitivity of early detection of gastrointestinal tumors (Song et al., 2020). The changes in intestinal microbiota suggested that regulating intestinal microbiota might be an interesting and promising target to lower toxins of uremic patients and thereby the incidence of cancer.

## Mechanisms of Paraneoplastic Kidney Injury

Previous studies found that there might be a higher risk of CKD incidence in cancer survivors than non-cancer patien ts (Shin et al., 2015). A large retrospective study on lymphoma patients also highlighted the high prevalence and mortality of CKD in lymphoma patients (Ubukata et al., 2018). And it has been indicated that CKD might be a prognostic factor for 5-year mortality of lung cancer patients, and might limit treatment options of lung cancer, leading to poor therapeutic effects and increased death risk (Wei et al., 2018).

### Solid Tumor-Related Kidney Disease

In 1966, a case of remission of nephrotic syndrome in a patient with colon cancer combined with nephrotic syndrome after surgery was reported (Lee et al., 1966), which was an early study linking cancer and nephrotic syndrome. The authors suggested that cancer might act as an antigen, producing antigen-antibody complexes that can be deposited along the glomerular basement membrane and anchored to the glomerular capillary basement membrane (Lee et al., 1966). In recent years, more cases of paraneoplastic nephropathy have been reported with clinical manifestations of hematuria and proteinuria, and many hypotheses and explanations have been proposed, including immune factors, tumor antigen deposition, antigen-antibody complex formation, vascular endothelial growth factor dysregulation and cancer-associated thrombotic microangiopathy, and mainly focusing on injury to the glomeruli and renal vessels (Eriguchi et al., 1998; Bacchetta et al., 2009; Lien and Lai, 2011).

Kidney biopsies from patients with palatal tumors combined with nephrotic syndrome showed the presence of carcinoembryonic antigens in glomerular deposits and their distribution was similar to that of immunoglobulins and their fractions to a certain degree (Borochovitz et al., 1982). Although researchers could find deposits of tumor antigens or antibodies in the glomeruli of such patients, the presence of neoplastic nephropathy was not necessarily associated with these deposits, as immune glomerular deposits without proteinuria were common in solid tumor-induced paraneoplastic nephropathy (Borochovitz et al., 1982; Bacchetta et al., 2009). Nevertheless, clinical experiences showed that when primary tumor disease was treated and in remission, part of these secondary renal injuries were even reportedly cured, as evidenced by a case of a patient with gastric cancer combined with nephrotic syndrome who maintained a cured state of renal disease 10 years after subtotal gastrectomy (Cantrell, 1969; Eriguchi et al., 1998), leaving this interdiscipline elusive but quite absorbing.

### Hematologic Tumor-Related Kidney Disease

Different from renal damage caused by solid tumors, hematological tumors might directly trigger renal injury and lead to secondary nephropathy through producing toxic metabolic substances. Under physiological conditions, free light chain (FLC) can be filtered through the glomerulus and then metabolized through proximal renal tubules (Sathick et al., 2019). The monoclonal FLC produced by multiple myeloma (MM) could have direct toxic effects on resident renal cells, leading to acute kidney injury (AKI) and CKD (Basnayake et al., 2011). Among them, monoclonal FLC caused the most common renal tubular injury, mainly characterized by monoclonal FLC deposition in proximal tubular epithelium or distal tubule lumen (Bridoux and Fermand, 2012). This might be related to the overproduction of FLC resulted from MM and saturation of the renal tubular transport system, which led to homotypic aggregation of FLC in proximal tubular epithelial cells to form intracellular crystals, or related to the role of FLC and Tamm Horsfall protein (THP) in the Henle loop associated to promote THP and LCs coaggregation to form cast (Sathick et al., 2019). In addition to those, metabolism of monoclonal FLCs in the proximal tubule might activate the redox-sensitive JAK2/STAT1 signaling pathway through redox signals, thereby generating a pro-inflammatory and pro-fibrotic environment in the kidney (Ying et al., 2019). Of note, the monoclonal FLCs could also promote STAT1-dependent release of HMGB1, an injury-associated molecular pattern, and increase the expression of TLR2, TLR4, and TLR6 as well as downstream inflammatory responses, all of which were important underpinnings of the injury that might migrate into CKD (Upadhyay et al., 2020). Some other cases reported that in Waldenstrom’s macroglobulinemia patients, electrodeposition of glomerular basement membrane could be seen under the electron microscope and immunofluorescence showed deposition of IgM and other immunoglobulins (Kaplan et al., 1976). It is hypothesized that Hodgkin’s lymphoma-related minimal change disease (MCD) might be related to excessive release of glomerular permeability factor IL-13 caused by immune imbalance (Kitai et al., 2015).

### Anticancer Therapy

Accumulating evidence indicated that anticancer treatments, including nephrectomy, chemotherapy, radiotherapy, molecular targeted therapy and immune checkpoint inhibitor, may also cause kidney injury, such as renal insufficiency, proteinuria, and hypertension (Shin et al., 2015; de Francisco et al., 2019). At present, it has been demonstrated that CKD patients undergoing nephrectomy had an increased risk of new-onset of cancer, especially in the presence of some comorbidities (Gazel et al., 2015; Ahn et al., 2018). In addition, nephron-sparing partial nephrectomy (PN) might also aggravate potential CKD, depending on the number of non-neoplastic renal parenchymal resections (McKiernan et al., 2002; Kotamarti et al., 2015). Reduction of healthy parenchymal volume after nephrectomy might lead to compensatory hypertrophy and over filtration of the remaining kidney, as 50% of the nephron was lost, which could result in glomerulosclerosis, proteinuria, elevation of the blood pressure and decrease in renal function in the long run (Dekkers et al., 2013; Kotamarti et al., 2015). Therefore, the preservation of healthy renal parenchyma and long-term follow-up was of great significance to prevent the occurrence of CKD after nephrectomy.

AKI had been recognized as one of the major reasons for CKD over the decades, which could be secondary to drug-induced nephrotoxicity (Black et al., 2018). Platinum-containing drugs commonly used in chemotherapy could mediate cytotoxic effects through nuclear and mitochondrial DNA damage and directly induce renal epithelial cell apoptosis (Shin et al., 2015). Among them, cisplatin-induced nephrotoxicity was widely accepted as a model of AKI (Shi et al., 2018). Previous study had shown that inhibition of NF-κB-mediated NLRP3/Caspase-1/GSDMD pyroptosis pathway might reduce cisplatin-induced AKI (Jiang et al., 2021). The experimental data of repeated low-dose cisplatin-induced AKI progression to CKD confirmed that unresolved injury and sustained activation of regulated necrosis pathways, rather than fibrosis, might promote the progression of cisplatin-induced AKI to CKD (Landau et al., 2019). Therefore, for cancer patients with CKD, especially patients with advanced or obvious ESKD, therapeutic drug monitoring, such as accurate assessment of the safety of anti-cancer drug doses for dialysis patients with cancer, was of prime importance if the start of this anti-tumor treatment was planned (Cosmai et al., 2016).

To date, molecular targeted therapy, which targeted characteristic changes of tumor cells, has increasingly attracted attention. Bevacizumab was one of the classical molecularly targeted drugs against vascular endothelial growth factor (VEGF), which has been used in treatment for various cancers such as metastatic colorectal cancer, non-small cell lung cancer and breast cancer (Zhu et al., 2007). A meta-analysis found that bevacizumab was associated with a significantly increased risk of proteinuria and hypertension in patients with metastatic breast, colorectal and renal cancer (Zhu et al., 2007). Removing VEGF from renal podocytes of conditionally gene knockout adult mice could lead to severe thrombotic glomerular injury, which suggested that glomerular injury in patients treated with bevacizumab might be due to direct targeting of VEGF by antiangiogenic therapy (Eremina et al., 2008). Many renal biopsies in patients treated with anti-VEGF drugs also confirmed glomerular micro-vessels were one of the main targets of injury (Lameire et al., 2011). In addition, blocking VEGF could also trigger endothelial dysfunction and influenced VEGF-associated vascular pathways (down-regulating vasodilatory pathways such as nitric oxide and prostaglandin I2, and up-regulating vasoconstriction pathway such as endothelin-1), resulting in increased afterload and hypertension (Fukasawa et al., 2014). Consequently, all drugs acting on the VEGF pathway might induce renal abnormalities, which were clinically manifested as hypertension, proteinuria, decreased GFR and thrombotic microvascular disease (Launay-Vacher et al., 2015).

Immune checkpoint inhibitor (ICI) was a new immunotherapeutic antitumor drug that played a role in blocking intrinsic downregulation of the immune system (Herrmann and Perazella, 2020). Pembrolizumab was a humanized monoclonal anti-programmed death 1 (PD1) pathway antibody that has been extensively investigated in numerous malignancies (Kwok et al., 2016). Pelaez Bejarano et al. showed a case of non-small cell lung cancer treated with Pembrolizumab who developed concurrent AKI, which was diagnosed with interstitial nephritis and finally progressed to CKD stage G2-G3, although Pembrolizumab was stopped immediately and hormone treatment was added (Pelaez Bejarano et al., 2021). *In situ* hybridization and immunohistochemical staining showed that mRNA and protein levels of programmed death ligand-1 (PD-L1) were expressed, though relatively low, in proximal tubules of normal kidney (Ding et al., 2005). Injecting anti-PD1 or anti-PD-L1 antibodies not only blocked PD-1 receptor, but also triggerred T cell proliferation and cytotoxic injury of the kidney (Dumoulin et al., 2020). Compared with classic acute tubulointerstitial nephritis patients, ICI-related interstitial nephritis showed similar renal histological manifestations, but there were potential differences in the development of pathological mechanisms, such as longer incubation period, lighter acute renal injury, and slower improvement of creatinine, etc (Draibe et al., 2021). Retrospective cohort study showed the incidence of CKD in 2,563 cancer patients treated with ICI for more than 1 year was approximately 6.34/100 person-years, indicating that CKD events were common in ICI therapy (Chute et al., 2022). Understanding the potential renal toxicity of these anti-cancer therapies would be of great significance to early detection and treatment for these patients and might improve diasease prognosis.

### Biomarkers in CKD Patients

In the presence of cancer risk, long-term and close follow-up observations, and cancer screening are needed to evaluate the renal function of these CKD patients. In addition to imaging examination, endoscopyand other screening methods, the monitoring of serum concentration of tumor markers has also been widely used for clinical diagnosis of cancer in recent years. However, diagnosis of cancer based on levels of these tumor biomarkers might be unreliable in CKD population, as increased serum concentration of some small molecular tumor markers has been oftern observed in these patients due to reduced glomerular filtration rate and metabolic disorder (Mikkelsen et al., 2017). Rani et el have shown that in advanced CKD patients (stages G4 and G5) without tumor the levels of carcinoembryonic antigen (CEA), human chorionic gonadotropin (HCG), carbohydrate antigen 199 (CA199) and carbohydrate antigen 153 (CA153) were significantly higher compared to the healthy controls (Rani et al., 2019). Additionally, elevation of some tumor markers might be non-tumour specific but associated with other comorbidities instead of cancer in CKD patients (Perkins et al., 2003). It was illustrated that CA153 and CA125 elevated in CKD patients without tumorigenesis but might be associated with concomitant active hepatitis C (Tzitzikos et al., 2010).

Nevertheless, there were studies indicating that serum levels of cytokeratin19 fragment (CYFRA21-1) might be involved in the development of epithelial cell carcinoma and chromogranin A (CGA) be associated with neuroendocrine tumor in CKD patients, and their reference limits might differ from those of healthy subjects (Mikkelsen et al., 2017). Another study found that CEA, squamous cell carcinoma (SCC), carbohydrate antigen 50 (CA50) and neuron-specific enolase (NSE) showed high false positive rates compared to normal reference values of the indicators in CRF patients and concluded that these values were unreliable in CRF patients, whereas carbohydrate antigen 125 (CA125), CA153, CA199, alpha-fetoprotein (AFP), prostate-specific antigen (PSA) and prostatic acid phosphatase (PAP) were still specific for assessing tumors in CRF with different cutoff values (Cases et al., 1991). Interestingly, Vitores et al. implicated that the decline of renal function seemed to show little impacts on the levels of serum CA724, suggesting that it might be of some significance of CA724 as an indicator for cancer diagnosis in CKD patients (González Vitores et al., 1999). And a recent study mentioned that prostate-specific antigen (PSA), alpha-fetoprotein (AFP), and *β*-human chorionic gonadotropin (β-HCG) might be reliable for cancer screening in dialysis patients, while total prostate-specific antigen (TPSA) and β2-microglobulin (β2-M) might be of some value in patients after renal transplantation (Amiri, 2016). The significance of some tumor biomarkers as indicators in CKD patients was summarized in Table 1 (Table 1). Notably, to date, whether the screening of traditional tumor markers can be used as cancer monitoring in CKD patients is still controversial and specific biomarkers for early screening cancer in these patients require further investigations.

**TABLE 1:** Tumor markers in CKD.

Tumor markers	CKD		Significance	References
	Non-dialysis	On dialysis		
CEA	H	H	Monitoring for malignancy in uremic patients might be unreliable	Tzitzikos et al., 2010; Cases et al., 1991; Rani et al., 2019
SCC	H	H	Monitoring for malignancy in uremic patients might be unreliable	Cases et al., 1991; Xiaofang et al., 2007
CA50	H	H	Monitoring for malignancy in uremic patients might be unreliable	Cases et al. (1991)
NSE	H	H	Monitoring for malignancy in uremic patients might be unreliable	Cases et al., 1991; Xiaofang et al., 2007
CA125	N or H	N	Monitoring for malignancy in dialysis patients might be unreliable	Cases et al., 1991; Mikkelsen et al., 2017
CA153	N or H	N or H	Monitoring for malignancy in uremic patients might be controversial	Cases et al., 1991; Xiaofang et al., 2007; Rani et al., 2019; Tzitzikos et al., 2010
CA199	N or H	N	Monitoring for malignancy in uremic patients might be comparatively reliable	Cases et al., 1991; Xiaofang et al., 2007; Rani et al., 2019; Mikkelsen et al., 2017
CA724	N	N	Monitoring for malignancy in dialysis patients might be reliable in dialysis patients	Xiaofang et al. (2007)
CYFRA21-1	H	—	Monitoring for malignancy in uremic patients might be possibly unreliable	Xiaofang et al., 2007; Mikkelsen et al., 2017
HCG	H	—	Monitoring for malignancy in uremic patients might be possibly unreliable	Rani et al. (2019)
PSA	N	N	Monitoring for malignancy in uremic patients might be possibly reliable	Cases et al. (1991)
AFP	N	N	Monitoring for malignancy in uremic patients might be reliable	Tzitzikos et al., 2010; Cases et al., 1991; Xiaofang et al., 2007
PAP	N	N	Monitoring for malignancy in uremic patients might be possibly reliable	Cases et al. (1991)
CGA	H	—	Monitoring for malignancy in uremic patients might be possibly unreliable	Mikkelsen et al. (2017)

H = high serum level; N = normal serum level.

## Conclusion

**FIGURE 1:**
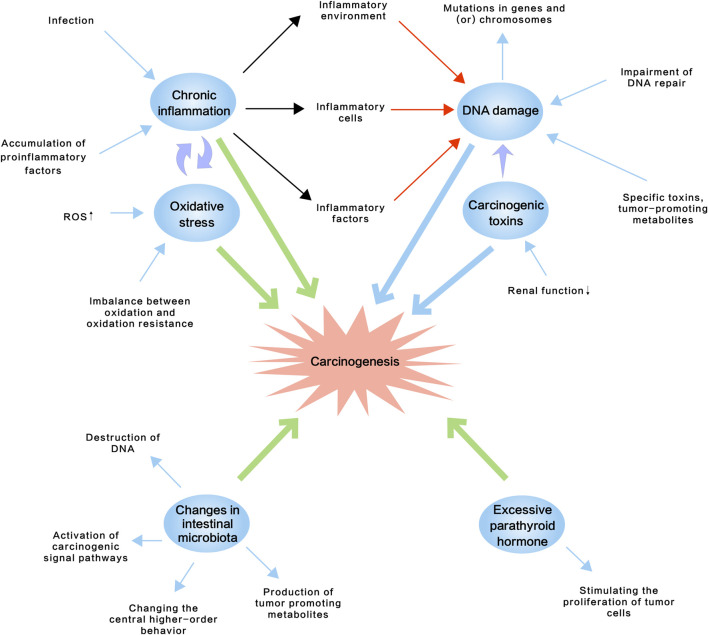
Schematic diagram of potential molecular mechanisms of the relatively high incidence rate of cancer in CKD, which might include chronic inflammation, accumulation of carcinogenic compounds, oxidative stress, impairment of DNA repair, excessive parathyroid hormone and changes in intestinal microbiota. ROS, reactive oxygen species.

**FIGURE 2:**
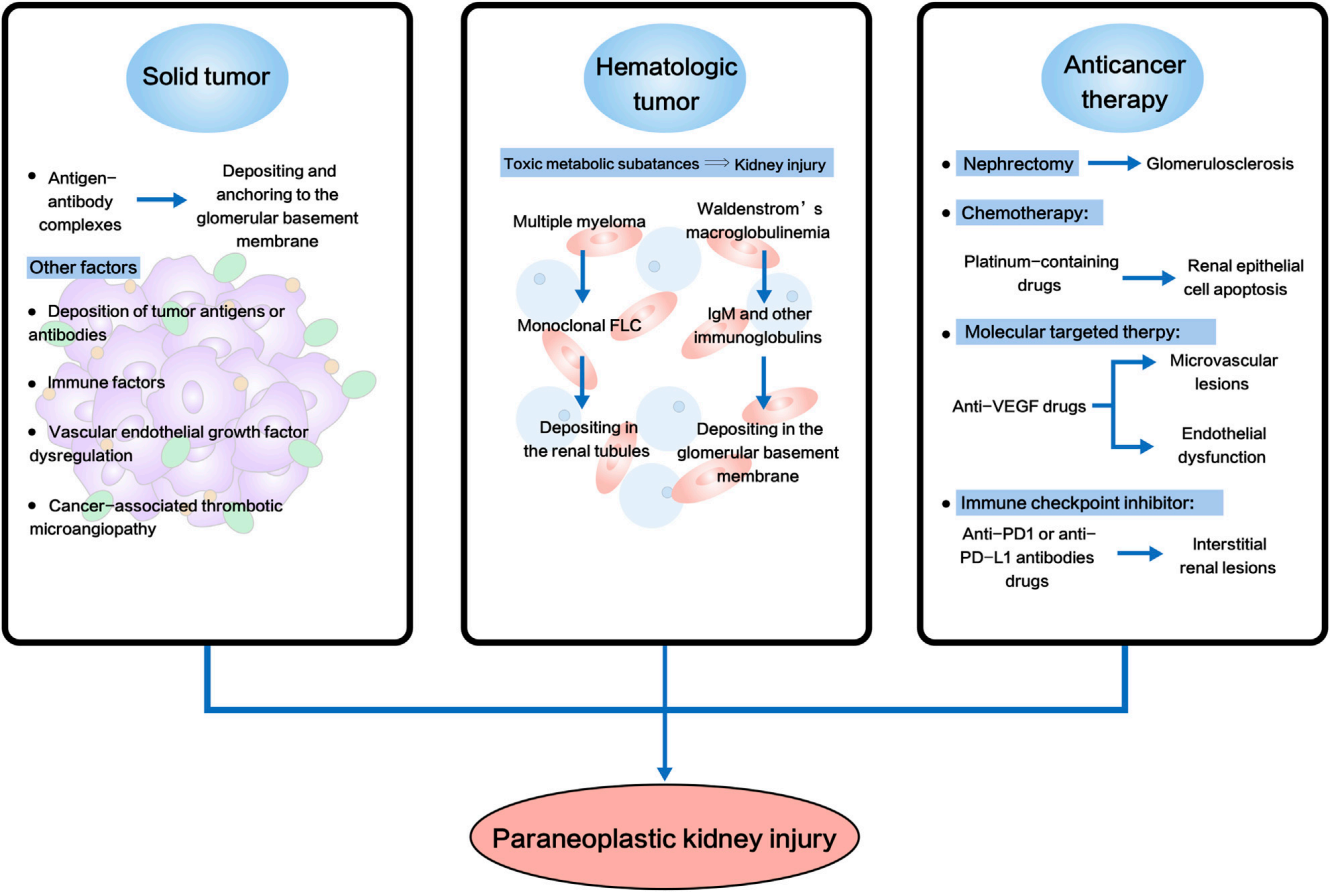
Schematic diagram of potential molecular mechanisms of paraneoplastic kidney injury. Paraneoplastic kidney injury was linked to solid tumor, hematologic malignancies and anti-cancer therapies. FLC, free light chain; VEGF, vascular endothelial growth factor; PD1, programmed death 1; PD-L1, programmed death ligand-1.

Recent advances in onco-nephrology have emphasized the necessity of understanding the high morbidity of cancer in CKD patients and paraneoplastic kidney injury, and the potential underlying mechanisms for both nephrologists and oncologists. In this review, we concluded from the literature that increased cancer incidence rate in CKD might be due to: 1) chronic inflammation, 2) accumulation of carcinogenic compounds, 3) oxidative stress, 4) impairment of DNA repair, 5) excessive parathyroid hormone, and 6) changes in intestinal microbiota (Figure 1; Table 2). And paraneoplastic kidney injury was linked to hematologic malignancies, carcinoma and anti-cancer therapies (Figure 2; Table 3). High risk of cancer in CKD implicated a regular cancer screening, including biomarker monitoring, imaging examination and endoscopy, might be of clinical significance for these populations for early detection and diagnosing and for improved prognosis. Nonetheless, the molecular mechanisms of CKD-associated tumorigenesis, and carcinoma- or anti-cancer therapy-associated kidney injury remain profoundly elusive, although for the past decades, the link with hematologic malignancies has been well established and there has been rapid progress in this interdisciplinary field. Further investigations into this field might open up new avenues for treating both CKD and cancer patients, facilitate improvements in therapeutic options and bring better patient outcome.

**TABLE 2:** Selected signaling pathways and transcription factors involved in CKD related to carcinogenesis.

Pathways/transcription factors	Animal model/cell analyzed	Indications	Results	References
ROS/Src/FAK	Human bladder cancer cells	Uremic toxin P-CS could induce bladder cancer cell migration and EMT through ROS/Src/FAK signaling pathway	Bladder cancer	Peng et al., 2020
Nrf2-NF-κB	*In vivo*: patients on hemodialysis (HD)	Oxidative stress in CKD patients resulted in a downregulation of the antioxidant effect of the Nfr2 system as well as an upregulation of oxidative regulation triggered by concomitant NF-κB activation, which might be associated with certain T cell lymphoma	T cell lymphoma	Pedruzzi et al., 2015; Giri and Aggarwal, 1998
	*In vitro*: RAW 264.7 macrophage-like cells			
ADAM-17/TGF-α/EGFR	Human proximal tubule epithelial cells	Activation of EGFR might indirectly regulate CCL-2 and CCL-5 expression to induce tumors	Prostate, gastric and colorectal cancers	Morgado-Pascual, et al., 2015; Yamaguchi et al., 2021
AHR	*In vivo*: CKD patients; mice modal of CKD	Uremic toxin might activate AHR in CKD to participate in pro-tumor effects	Hodgkin’s lymphoma, chronic lymphocytic leukemia, adult T-cell leukemia, and cancers of the breast, head and neck, brain, kidney, lung, pancreas, and gastrointestinal tract	Dou et al., 2018; Wang et al., 2020b
Wnt/β-catenin	CKD patients	Inflammatory/oxidative processes are accompanied by activation of the Wnt/β-catenin signaling pathway, and the activated Wnt/β-catenin signaling pathway is involved in the development and progression of solid tumors and hematologic malignancies	Hepatocellular carcinoma and multiple myeloma	Chen et al., 2017; He and Tang, 2020; Hu and Hu, 2018

**TABLE 3:** Selected signaling pathways and transcription factors involved in chronic kidney injury in cancer patients.

Pathways/transcription factor	Animal model/cell analyzed	Indications	Results	References
VEGF	Adult mice with renal podocytes deleted with VEGF	Glomerular injury in patients treated with bevacizumab might be due to direct targeting of VEGF by antiangiogenic therapy	Thrombotic glomerular injury	Eremina et al. (2008)
NLRP3/Caspase-1/GSDMD	Vitamin D receptor knockout mice	NF-κB could mediate NLRP3/Caspase-1/GSDMD pathway of pyroptosis pathway to increase cisplatin-induced AKI.	Apoptosis and necrosis of the renal tubular epithelial cells	Jiang et al. (2021)
	Vitamin D receptor knockout human tubular epithelial cells			
JAK2/STAT1	Human proximal tubular epithelial cells	Monoclonal FLCs produce pro-inflammatory and pro-fibrotic events in proximal tubules leading to CKD through activation of the JAK2/STAT1 pathway	Renal tubular interstitial disease complicated by proximal tubular injury	Ying et al. (2019)
STAT1/HMGB1/TLR	Human kidney proximal tubular epithelial cells	The activation of TLR was involved in FLC-mediated renal inflammation	Renal tubular interstitial disease	Upadhyay et al. (2020)
	Stat1 knockout mice			
